# Gleanings Abroad

**Published:** 1854-07

**Authors:** 


"GLEANINGS ABROAD."—NAPLES, *June 23d*, 1854. BY AN EX-EDITOR. *Mr. Editor*:—Owing to the haste in which I drew up my last No., on the eve of my departure from Paris, I omitted to speak of sundry little matters which may perhaps interest your readers. Let us, then, go back to *Naples*. On arriving off the port, at 7 A. M., we were visited by the *Sanitary Commission*, a very thick-headed, savage-looking trio, who held a parley with our captain, at two oars' length from the steamer. After receiving all the necessary intelligence, they took their departure, to deliberate upon and decide our fate. In the mean time, we were quite uneasy as to the prospects of our being ordered to perform quarantine for ten days at the island of Nisida, opposite Puzzuoli. Although two weeks had elapsed since our departure from Paris, such is the dread of cholera in this region, and such the besotted ignorance and old fogyism of the high functionaries, that our position was anything but agreeable. However, in about two hours, we were relieved from our suspense, and allowed to land. Eighteen years previously, I had made the acquaintance of these same *old fogies*, and had then been condemned to suffer a quarantine of forty-eight hours, because, forsooth, we had touched at Genoa, and because that a vessel from New Orleans had arrived at Genoa in the *month of February;* the aforesaid port of New Orleans having, in the *summer previous*, been affected with yellow fever. This is a *fair specimen of progress* in this benighted country, where phthisis is yet considered contagious, where dying patients, or those supposed to be about to die, are transferred to the "dying ward," and where the miracle of the blood of St. Januarius liquifying is still annually performed and believed in; it is, in fact, essential to the preservation of good order, and to the stability of the government. In this same Naples may be nightly witnessed the interment of the poorer classes, as if they were cats or dogs. The dead-carts arrive at the Campo Santo at eight o'clock, one of the three hundred and sixty-five pits are opened, and bodies which have been collected during the previous twenty-four hours are thrown out, unshrouded and coffinless, into this Golgotha. The spectacle is too shocking and revolting even for medical eyes. Yet, while man is so vile, is Naples a terrestrial paradise, "situated on the verge of Elysium, enjoying all the advantages of land and water, on the confines of earth and ocean." I found the *climate* variable, and dependent on the direction of the wind. So late as the 28th of March, an overcoat was necessary, particularly when riding; there was also recent snow upon Vesuvius and the mountains adjacent; and when the sirocco blew from the coast of Africa, the effect was enervating, depressing both mind and body. When the tramontana comes down from the Appenines, the air is chilling cold; and invalids find great difficulty in keeping themselves comfortably warm, the doors and windows being very open, and fires a great rarity. The promontory of Sorento, across the bay, is equable in temperature, and therefore preferred by pulmonary invalids. The *physique* of the Neapolitans is admirable, more especially that of the men, the women having but little beauty to spare; the physiognomy is Grecian. The *lazaroni* (lazy-roonies) are walking Apollos, in grace and action perfect types of man. Their diet is exclusively vegetable and farinaceous, maccaroni being to them the staff of life. I am sorry that I cannot say much for their *morality*, as the great mass of them are either *pimps*, *beggars*, *or thieves.* So adroit are they at stealing pocket-handkerchiefs, that it is usual for travelers to carry this article of the toilette in their hats, or else not to trust themselves on the *pavé* at all. The streets are narrow lanes, with beams stretched across, to keep the houses from tumbling together, filthy and offensive to the last degree: the Toledo and the Chiaja are exceptions in this respect. The *mortality* in Naples is very large, *one twenty-eighth* part of the population being annually carried off. This may be attributed, in part, to moral and political causes, rather than to climate; I found, on inquiry, that phthisis was very common. To the medical traveler, a visit to the *Muséo Borbinico* is intensely interesting. He here sees a complete epitome of Roman life, in the vast collection of objects from Pompeii and Herculaneum. Not to enumerate any that are not of professional interest, he will here see the drugs of the apothecary, the instruments of the surgeon and accoucheur, and even the sign, painted in red letters, indicating the name and profession. I saw *lancets*, *probes*, *scalpeli*, *sounds*, *catheters*, *dressing-forceps*, *cautery irons*, *cupping apparatus*, all made of bronze, with the cutting edges hardened by a peculiar process now unknown; also two *specula uteri*, one with two valves like Ricord's, another with three valves, opening by a screw ingeniously contrived to force open the valves. This last was remarkably well fabricated, and is as perfect as when first made. Here are also *gilt pills*, *boluses*, and any quantity of *apothecariy's*

*ware.* To me, the most interesting relic in the whole collection was *the skull of the faithful sentinel*, that was found at the gate of Pompeii, at his post; his bronze helmet is *in capite.* There are two other skulls in this collection, being those of two culprits, who were found with "their feet fast in the stocks." The ashes of the wife of Diomede are here preserved, as is also the bone of the ring finger, with the gold ring attached. A *curious ring*, in fine gold, is here shown, with a *Phallus* engraved upon it, beautifully executed. This sort of ring was worn by barren women, in hope of becoming fertile.

In the apartment of the "*Oggetti riservati*," which is now a sealed room and accessable, I saw, at my first visit in 1836, all those objects which, from their peculiar character or their *indecency*, were not suited for the public gaze; such as ladies' work boxes or flower-stands with obscene figures upon them, necklaces, earrings, bracelets and amulets of the same character, steelyards, and even a *sarcophagus* covered with the grossest emblems of obscenity and crime. Never shall I forget the comical expression of the face of the guide who was descanting upon the individual merits of these works of art; pointing to a vase in basso relievo, representing the act of generation as performed by dogs, he exclaimed, "Voila, Messieurs, les chens, toujours malheureux!!" This was Roman civilization; these are relics of the days of Virgil and Cicero, of Mæcenas and Horace, of Varro and Hortensius; this the Augustan age of Heathenism. In the city of Pompeii, whence most of these objects were procured, one sees yet, upon the walls of two houses, a relievo representation of the god *Priapus:* whether these were for the residence of public women or not, is a disputed point among antiquarians, but the probabilities are in favor of their having been so appropriated. The temple of Æsculapius, and also of Mercury, are in good preservation; an apothecary's shop has for its sign a serpent devouring a pine-apple, emblematical of Prudence triumphing over Death; it is numbered, and the name of the proprietor is on the hall in red paint. The streets are narrow, the houses have low ceilings, with an open court in the center, a covered portico, into which open several small apartments, with no ventilation except through the doors. The house of Diomede, the largest yet discovered, has three stories, but two of them are under ground; *twenty-eight skeletons* were found in this house, two of them, that of Diomede and his slave, being found very near the door, running away, the former with a bag of money in his hand; his wine-cellars are in perfect preservation, and were well filled; it is situated hard by the Herculaneum Gate. Only one-fifth part of the city is uncovered by the excavations, and as these are still progressing, we may expect still more interesting discoveries, and may yet find a complete *armamentum chirurgicum* of some Pompeiian Valentine or Sir

Astley. The *Grotio del Cane* on the Lake of Agnano, two miles from Naples, is, to the medical philosopher, worth a visit. The cave is on a hill side, the floor of the cave being of a sandy loam; it is kept closed by a door, when not in exhibition. On approaching the cave, the guides demand whether you will see the "*sperimento:*" the bargain being made, which, in our case, was for four carlini, a terrier dog is taken and laid down on the floor of the cave with the door open; he is held down at first for a few seconds, when he becomes insensible, the respiration very labored, with discoloration of the lips, upturning of the eye, and frothing of the mouth; after one and a-half minutes he is taken out and thrown upon the green sward, where he soon recovers himself, and is about as lively as before. Indeed, I was surprised at this rapid recovery from the effects of the poison. *Three minutes' inhalation* of the gas is fatal. The stream of carbonic acid gas is about twelve inches in depth, and can be easily dipped up in the hand or hat, having the characteristic pungent odor; the extinguishing of the torch is beautifully shown, and the line of gas is very level throughout. Near by is the Grotto d'Ammonia, with deposits of the carbonate on the sides of the excavation, and a very powerful odor of carbonate of ammonia. On the opposite side of the road are the hot baths of St. Germano, much used in rheumatism; they are open at the top, as the vapor is too hot to be respired in a close room. On returning from Baia, we visited the *hot baths of Nero* on the sea-side, half-way up the rocky bluff, and extending for some distance into the calcareous tufa; hot vapor is seen escaping by the roof of the cave; the guide took a pail with an egg in it, and went down; after two minutes he returned panting, and dissolved in sweat; the egg was overdone. The present guide has been on duty for two years. I inquired for my old acquaintance, his predecessor; but he had *been boiled out of existence* some time since. The miserable appearance of the present guide convinced me that he could not take six and eight vapor baths a day without great detriment. These baths are much resorted to in summer by gouty and rheumatic invalids. Near Puzzuoli are other hot baths. Indeed the whole line of coast, from Misenium to Vesuvius, abounds in hot springs, &c., occurring in the tufa rock. Above Puteoli or Pozzuoli, on a high hill, is the *Soltafara*, a crater of a volcano now nearly extinct, here are extensive alum and sulphur works; the ground has a peculiarly hollow sound when struck by a falling body. At one extremity is a cavern whence the hot vapor rushes out with a noise like the escape of steam from a large boiler; this phenomenon is almost constant, and only ceases when Vesuvius, ten miles off, is in eruption. On descending to Pozzuoli, we were surrounded by *priapus merchants*, in brisk competition; these Priapi are in bronze and well made, with a small ring for suspension round the neck, or to a watchguard. Whether they are now sold as *charms against barrenness*, or as *obscene objects*, I am not prepared to determine; certain it is, that they are offered indiscriminately to *ladies* as well as gentlemen. It must also be borne in mind, that this occurs in the city of Puteoli, where the great apostle to the Gentiles landed on his way to Rome. A sermon from such authority would surely not be out of place at the present day. This exhibition is only one of the many relics of heathenism now met with in this region. I have done with Naples.

Yours,

—*N. Y. Medical Times.* J. G. ADAMS.

